# Cilia-related gene signature in the nasal mucosa correlates with disease severity and outcomes in critical respiratory syncytial virus bronchiolitis

**DOI:** 10.3389/fimmu.2022.924792

**Published:** 2022-09-23

**Authors:** Clarissa M. Koch, Andrew D. Prigge, Leah Setar, Kishore R. Anekalla, Hahn Chi Do-Umehara, Hiam Abdala-Valencia, Yuliya Politanska, Avani Shukla, Jairo Chavez, Grant R. Hahn, Bria M. Coates

**Affiliations:** ^1^ Department of Medicine, Northwestern University, Chicago, IL, United States; ^2^ Department of Pediatrics, Northwestern University, Chicago, IL, United States; ^3^ Ann & Robert H. Lurie Children’s Hospital of Chicago, Chicago, IL, United States

**Keywords:** RSV, nasal mucosa, RNA-Seq, bronchiolitis, pediatric critical care

## Abstract

**Background:**

Respiratory syncytial virus (RSV) can cause life-threatening respiratory failure in infants. We sought to characterize the local host response to RSV infection in the nasal mucosa of infants with critical bronchiolitis and to identify early admission gene signatures associated with clinical outcomes.

**Methods:**

Nasal scrape biopsies were obtained from 33 infants admitted to the pediatric intensive care unit (PICU) with critical RSV bronchiolitis requiring non-invasive respiratory support (NIS) or invasive mechanical ventilation (IMV), and RNA sequencing (RNA-seq) was performed. Gene expression in participants who required shortened NIS (</= 3 days), prolonged NIS (> 3 days), and IMV was compared.

**Findings:**

Increased expression of ciliated cell genes and estimated ciliated cell abundance, but not immune cell abundance, positively correlated with duration of hospitalization in infants with critical bronchiolitis. A ciliated cell signature characterized infants who required NIS for > 3 days while a basal cell signature was present in infants who required NIS for </= 3 days, despite both groups requiring an equal degree of respiratory support at the time of sampling. Infants who required invasive mechanical ventilation had increased expression of genes involved in neutrophil activation and cell death.

**Interpretation:**

Increased expression of cilia-related genes in clinically indistinguishable infants with critical RSV may differentiate between infants who will require prolonged hospitalization and infants who will recover quickly. Validation of these findings in a larger cohort is needed to determine whether a cilia-related gene signature can predict duration of illness in infants with critical bronchiolitis. The ability to identify which infants with critical RSV bronchiolitis may require prolonged hospitalization using non-invasive nasal samples would provide invaluable prognostic information to parents and medical providers.

## Introduction

Respiratory syncytial virus (RSV) is a ubiquitous, single-stranded RNA virus that is the leading cause of bronchiolitis in children. Most RSV infections are mild, with symptoms confined to the upper respiratory tract. However, in a subset of children, RSV can cause severe, life-threatening lower respiratory tract disease (1, 2). While certain predisposing conditions such as young age, prematurity, and chronic medical conditions increase the risk of severe RSV infection, most cases requiring medical attention occur in healthy children (3, 4). Notably, up to 2% of children less than one year of age and infected with RSV require hospitalization (5). In children less than 5 years of age, RSV is responsible for ~25% of acute lower respiratory tract infections (LRTI), 33.1 million hospitalizations, and 55-199,000 deaths per year worldwide (6). Currently, there is no predictive tool to identify which infants with RSV bronchiolitis admitted to the pediatric intensive care unit (PICU) will recover quickly and which infants will require prolonged respiratory support.

The respiratory epithelium is composed of a diverse group of cell types with distinct functions. The primary cell types in the nasal mucosa include ciliated cells, secretory cells, and basal cells (7). Ciliated cells facilitate the clearance of mucus, known as mucociliary clearance, through the rhythmic beating of cilia. Secretory cells, including goblet cells, produce mucus to trap debris and pathogens. Basal cells, located on the basement membrane, are the stem cells of the respiratory epithelium. They regenerate the epithelium following injury through proliferation and differentiation into other epithelial subtypes. Ciliated cells are the primary cell type infected by RSV (8). The binding of the RSV-F protein to insulin-like growth factor 1 receptor (IGF1R) results in protein kinase C zeta (PKCζ)-mediated nucleolin (NCL) recruitment to the cell surface. NCL facilitates membrane fusion, allowing for viral entry (9, 10). Cells infected with RSV become dysmorphic and dysfunctional, losing their ability to facilitate mucociliary clearance. Dysmorphic epithelial cells protrude and are shed into airways, contributing to the obstructive physiology in infants with RSV bronchiolitis (11–13).

The host response to RSV is the key determinant of disease severity and clinical outcomes. Studies evaluating cytokine levels in nasal aspirates of children with RSV suggest proinflammatory cytokines may contribute to the severity of illness (14–16). Similarly, upregulation of neutrophil activation genes in the nasal mucosa during RSV infection is associated with symptomatic disease (17–19). Comparisons of the nasal transcriptional response between RSV-infected and uninfected infants demonstrate upregulation of interferon and innate immune genes during RSV infection (17). The impact of the interferon and innate immune response to RSV on clinical outcomes during critical infections in infants is unclear.

Improved understanding of the host response to RSV infection may help with prognostication, identification of therapeutic targets, and stratification of high-risk patients for interventions. Unfortunately, accurate phenotyping of LRTI in infants is challenging due to limited access to samples from the lower airways. Although immune responses in the blood have been associated with outcomes, they do not reliably reflect immune responses at the site of infection during viral LRTI, leaving their mechanistic role in the pathogenesis of infection unclear (17, 20, 21). Notably, nearly 90% of genes in the healthy infant nasal transcriptome mirror those identified in the lower airways of healthy adults, suggesting gene expression in the nasal mucosa in infants may provide insight into the transcriptional responses of the lower airways (22, 23).

We performed RNA sequencing (RNA-seq) to interrogate the transcriptional response to RSV in the nasal mucosa of infants with critical RSV bronchiolitis to identify gene signatures associated with severity of illness and outcomes. We identified a ciliated cell gene signature correlated with hospital and PICU length of stay in infants sampled within three days of PICU admission. Epithelial gene signatures were also associated with prolonged respiratory support in critical RSV bronchiolitis. Our data suggest that there are distinct transcriptional changes in the nasal mucosa near the time of PICU admission in children who will recover from critical RSV quickly and children who will require prolonged respiratory support.

## Methods

### Study population

Approval for this study was obtained from the Institutional Review Board at Ann & Robert H. Lurie Children’s Hospital of Chicago (Chicago, IL, USA). Informed consent was obtained from parents/guardians of all participants. Participants were recruited during two viral respiratory seasons: December 2017 thru April 2018 and December 2019 thru March 2020. A subset of the participants recruited December 2019 thru March 2020 were included in a previous study (24). Medical records of children admitted to the PICU at Ann & Robert H. Lurie Children’s Hospital of Chicago were screened for inclusion criteria: age <2 years, polymerase chain reaction (PCR) or rapid antigen-positive RSV infection, and symptoms of pulmonary parenchymal disease including any of the following: 1) rales, wheezing, retractions, tachypnea, or any form of respiratory distress on exam, or 2) a supplemental oxygen requirement to maintain transcutaneous oxygen saturation levels above 95%, or 3) chest radiograph with areas of atelectasis, infiltrates, or hyperinflation. Exclusion criteria included: evidence of superimposed bacterial infection and any significant medical comorbidities, including: lung disease, hemodynamically significant cardiac disease, immunodeficiency, malignancy, neurologic disorders increasing the risk of aspiration, genetic and metabolic conditions, and preterm birth prior to 35 weeks gestation.

### Patient enrollment

We enrolled 53 eligible participants in the study. Two participants withdrew consent prior to sample collection. After assessment of RNA quality, 17 samples were excluded due to low quality or inadequate quantity of RNA for analysis. One patient was excluded for determination of superimposed bacterial infection. A cohort of 33 participants was used for the analysis.

### Clinical data

Clinical data were managed using REDCap electronic data capture tools hosted at the Northwestern University Clinical and Translational Sciences Institute (25). Hospital length of stay was determined from admission and discharge times. PICU length of stay was calculated from admission and transfer times. Duration of non-invasive respiratory or mechanical ventilatory support was approximated in 8-hour intervals. Non-invasive support was defined as high flow nasal cannula or positive pressure ventilation provided through a nasal interface.

### Nasal curettage and RNA isolation

Nasal mucosa samples were obtained by mucosal scrape biopsy of the inferior turbinate using a sterile plastic curette (Rhino-Pro curette, Arlington Scientific) as previously described (23). Nasal mucosa curettage was performed by trained study personnel on all participants for consistency in sample collection. Three single-pass scrapings per nare were performed on each subject. The curette tips were placed immediately into RNase-free tubes containing 350 uL of RLT plus buffer (Qiagen, Germantown, MD, U.S.A.) supplemented with 2-mercaptoethanol, vortexed vigorously, and stored at -80°C until RNA extraction using Qiagen RNeasy extraction kits.

### RNA sequencing and analysis

RNA quantity and quality were assessed using TapeStation 4200 High Sensitivity RNA tapes (Agilent), and RNA-seq libraries were prepared from 1 ng of total RNA using SMARTer Stranded Total RNA-seq Kit v2 (Taka Bio). After quality control of cDNA libraries using TapeStation 4200 High Sensitivity DNA tapes (Agilent), dual indexed libraries were pooled and sequenced on a NextSeq 500 instrument (Illumina), 75 cycles, single-end, to an average sequencing depth of 10M reads. FASTQ files were generated using bcl2fastq (Illumina). Viral RNA was detected using the RSV/S2 ts1C (GCF_000856445.1) genome. To facilitate reproducible analysis, samples were processed using the publicly available nf-core/RNA-seq pipeline version 1.4.2 implemented in Nextflow 19.10.0 using Singularity 3.2.1-1 with the minimal command nextflow run nf-core/rnaseq -r 1.4.2 –singleEnd -profile singularity –reverseStranded –three_prime_clip_r2 3 (26–28). Briefly, lane-level reads were trimmed using trimGalore! 0.6.4 and aligned to the hybrid genome described above using STAR 2.6.1d (29). Gene-level assignment was then performed using feature Counts 1.6.4 (30).

Pairwise comparisons were run using edgeR to identify differentially expressed genes between groups. Analyses to identify enriched Gene Ontology (GO) terms in target lists of up- or downregulated genes compared to total background were run using GOrilla. Gene set enrichment analysis (GSEA) was run using the weighted, pre-ranked setting with ranked log2FC values reported by edgeR as input (31). Average normalized expression of IFN response genes was calculated by combining the Hallmark Pathway lists for IFNα and IFNγ due to significant overlap between the two pathways. Specifically, 75% of the Hallmark gene set genes for the IFNα response are also part of the IFNγ response gene set.

### Weighted gene co-expression network analysis (WGCNA)

WGCNA was performed using WGCNA version 1.69 with default settings unless otherwise specified (32). To focus on the early host response to RSV, only samples collected within 3 days of ICU admission were used for analysis (n=28). Highly variable genes (HVGs) among participant samples were identified and normalized reads (CPM) for HVGs were used as input for WGCNA. Using the pick Soft Threshold function, we empirically determined a soft threshold of 7 to best fit the network structure. A minimum module size of 30 genes was chosen. Module eigengenes were then related to patient and sample metadata using biweight midcorrelation. Module GO enrichment was then determined using GOrilla (33).

### Deconvolution of bulk RNA-seq signatures

Deconvolution of bulk RNA-seq data was performed using AutogeneS v.1.0.3 (34). We used a single-cell RNA-seq dataset from Ordovas-Montanes et al. (35) containing data from human nasal epithelial and immune cell subsets, to train the AutoGeneS model. AutoGeneS selects cell type-specific genes by simultaneously minimizing correlation between and maximizing the distance between cell type-specific average gene expression profiles. This model was then applied to our bulk RNA-seq data to estimate relative cell type abundance.

### Statistical analysis

All clinical variables were plotted and assessed using the Shapiro-Wilk Test to assess for normality. Non-parametric continuous data were summarized using medians with interquartile ranges and compared using Mann-Whitney U or Kruskal-Wallis testing. Categorical data were summarized by percentages and compared using Fisher’s exact test. When performing multiple comparisons, the Benjamini-Hochberg procedure was used for false discovery rate correction. Data were considered statistically significant at P-adjusted <0.05. Statistical analyses were performed using Graphpad Prism version 9.1.1 and R version 4.0.3. Data were visualized using ggplot2 version 3.3.2 and Graphpad Prism.

## Results

### Participant cohort

**Figure 1:**
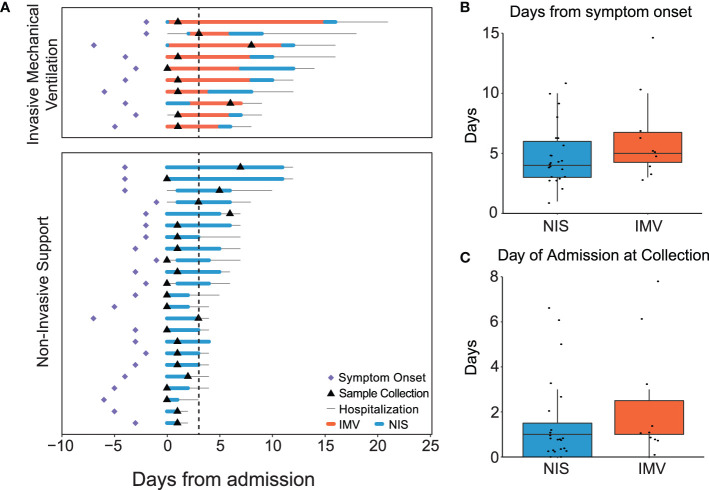
Clinical course of participants. **(A)** Timeline of symptom onset (diamond), sample collection (triangle), hospital stay (thin line), duration of non-invasive support (blue line), and duration of invasive mechanical ventilation (orange line) in all RSV infected patients (n=33). Samples collected within 3 days of admission (dotted line). **(B)** Median days from symptom onset to sample collection in mechanically ventilated and non-invasively supported participants. Comparison of medians performed using the Mann-Whitney U test. **(C)** Median days from admission to sample collection in mechanically ventilated and non-invasively supported participants. Comparison of medians performed using the Mann-Whitney U test. All p-values were adjusted using Benjamini-Hochberg FDR correction. Differences were not significant (p-adjusted>0.05) unless noted. RSV, respiratory syncytial virus; NIS, Non-invasive support; IMV, invasive mechanical ventilation; FDR, false discovery rate.

The clinical course of the participants is summarized in **Figure 1A**. Ten participants were supported with IMV, while 23 received NIS. The time from reported symptom onset to sample collection was similar between participants receiving IMV and NIS (**Figure 1B**). The timing of sample collection relative to admission day was also similar between the two groups, ranging from 0 to 8 days (**Figure 1C**). Samples were collected within 3 days of admission for 28 of the 33 participants (85%). The infants who received IMV were similar in age, gestational age at birth, race, and ethnicity to those that received NIS (**Table 1**). A larger proportion of infants who received IMV were female compared to infants who received NIS. As expected, the participants who received IMV had a longer length of stay and duration of respiratory support than the participants receiving NIS.

**Table 1:** Description of RSV participants.

	RSV	NIS	IMV	P-Adj
**n**	33	23	10	
**Median Age (mon) [IQR]**	2.0 [1.8-5.0]	2 [1.6-4.5]	3 [1.8-5.0]	0.74
**Gest Age <35 wks at Birth (%)**	1 (3.0)	0 (0.0)	1 (10.0)	0.43
**Sex**				
Male (%)	17 (51.5)	16 (70.0)	1 (10.0)	0.006
Female (%)	16 (48.5)	7 (30.4)	9 (90.0)	
**Race**				
White (%)	17 (51.5)	13 (56.5)	4 (40.0)	0.74
African American (%)	4 (12.1)	3 (13.0)	1 (10.0)	
Asian (%)	2 (6.1)	1 (4.3)	1 (10.0)	
Other (%)	10 (30.3)	6 (26.1)	4 (40.0)	
**Ethnicity**				
Non-Hispanic (%)	23 (70.0)	17 (74.0)	6 (60.0)	0.55
Hispanic (%)	10 (30.3)	6 (26.1)	4 (40.0)	
**Sample Collection**				
Day of Illness (d) [IQR]	4.0 [3.0-6.0]	4.0 [3.0-6.0]	5.0 [4.3-6.8]	0.43
Day of Admission (d) [IQR]	1.0 [0.0-2.0]	1.0 [0.0-1.5]	1.0 [1.0-2.5]	0.43
**Outcomes**				
Median ICU LOS (d) [IQR]	5.6 [3.4-8.8]	4.0 [3.0-5.6]	9.8 [7.8-12.0]	0.0002
Median Hospital LOS (d) [IQR]	6.7 [4.1-11.5]	5.0 [3.8-6.7]	13.2 [9.5-15.9]	0.0002
Median Respiratory Support Duration [IQR]	4.7 [3.0-7.3]	3.0 [2.7-4.8]	8.5 [7.3-11.4]	0.0004
Median Mechanical Ventilation Duration [IQR]	6.3 [4.3-7.6]	–	6.3 [4.3-7.6]	

RSV, Respiratory Syncytial Virus; NIS, non-invasive support; IMV, invasive mechanical ventilation; mon, months; IQR, interquartile range; Gest, gestational; wks, weeks; ICU, intensive care unit; LOS, length of stay; d, days.

### Interferon response correlates with viral reads, but not with clinical outcomes, in critical RSV bronchiolitis

**Figure 2:**
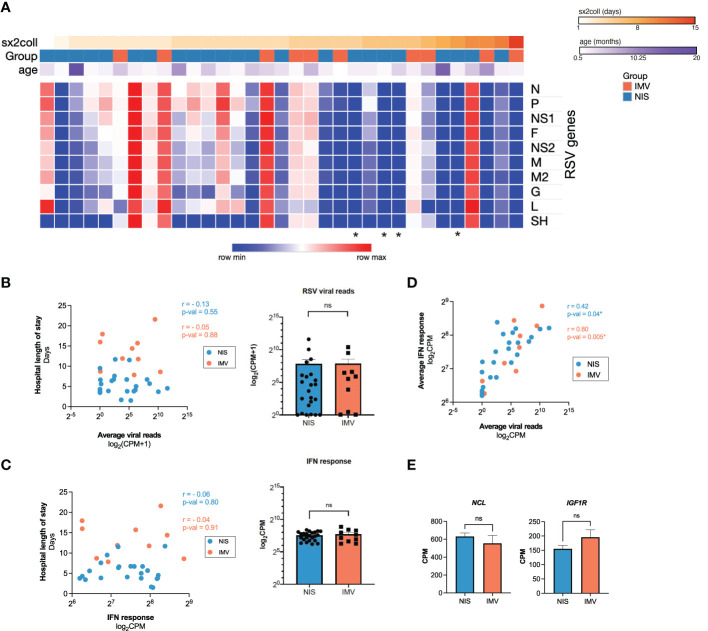
Interferon response correlates with viral reads, but not with clinical outcome in critical RSV bronchiolitis. **(A)** Heatmap of normalized reads for RSV/S2 ts1C detected in RNA-seq data for all participant samples (n = 33). Normalized reads (CPM) are shown. **(B)** Averaged RSV viral reads did not correlate with hospital length of stay in participants on NIS or IMV support. Log-transformed normalized counts (CPM) are shown. Pearson correlation coefficient (r) and p-value are shown. **(C)** Average normalized expression of IFN response genes (combined Hallmark pathway lists for IFNα and IFNγ response) did not correlate with hospital length of stay in participants on NIS or IMV support. Log-transformed normalized counts (CPM) are shown. Pearson correlation coefficient (r) and p-value are shown. **(D)** Average normalized expression of IFN response genes correlates with averaged RSV viral reads. **(E)** Expression (CPM) of RSV entry factors *NCL* and *IGF1R* genes: N, Nucleoprotein; P, Phosphoprotein; NS1, non-structural protein 1; F, Fusion protein; NS2, non-structural protein 2; M, Matrix protein; M2, Matrix protein; G, Glycoprotein; L, Large protein; SH, Small hydrophobic protein; NIS, non-invasive support; IMV, invasive mechanical ventilation; IFN, interferon; CPM, counts per million; NCL, nucleolin; IGF1R, insulin-like growth factor-1 receptor; *, no RSV reads detected; ns, not significant.

Viral load and the antiviral interferon response are two mechanisms hypothesized to contribute to outcomes in viral infections (36–42). To assess viral load across the spectrum of illness in our cohort, we measured the expression of RSV genes in all participants (n=33) (**Figure 2A**). The average expression of RSV viral reads detected in the nasal mucosa did not correlate with the duration of hospitalization. Similarly, the average viral reads in participants requiring NIS and IMV were not different (**Figure 2B**). Next, we assessed for an association between the interferon response and clinical outcomes. The averaged normalized expression of interferon response genes did not correlate with hospital length of stay and was similar between participants requiring NIS and IMV (**Figure 2C**). In contrast, we observed a positive correlation between viral reads and the interferon response (**Figure 2D**). RSV entry into epithelial cells is facilitated by the cell surface proteins NCL and IGF1R (9, 10). Thus, we examined their expression in our cohort. There was no difference in the expression of *NCL* or *IGF1R* between participants receiving NIS and IMV (**Figure 2E**). Altogether, these data suggest that expression of viral entry factors, viral load, and the local interferon response are not associated with severity of illness or outcomes in critical RSV bronchiolitis.

### Early transcriptional signatures associated with outcomes in critical RSV bronchiolitis enrich for ciliated cell genes

**Figure 3:**
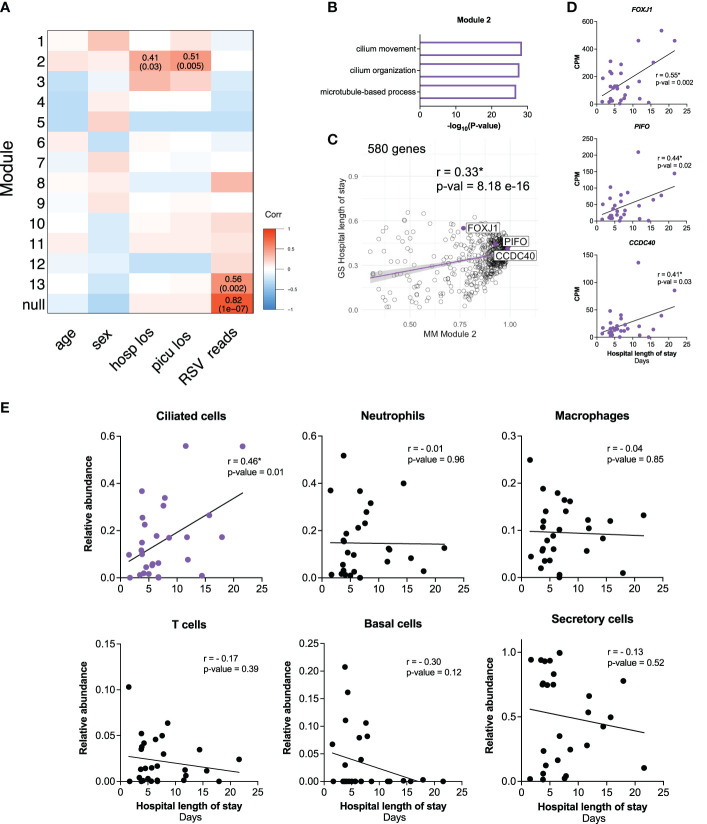
Early transcriptional signatures associated with outcomes in critical RSV bronchiolitis enrich for ciliated cell genes. **(A)** 2409 HVGs for early participant samples (n=28) were analyzed using WGCNA. Module-trait relationships were identified for 13 modules. Modules 1-13 contain 37, 580, 92, 160, 229, 133, 250, 45, 187, 308, 62, 152, 95 respectively. 79 genes that did not fall into any module were assigned to the null module. **(B)** GO biological process enrichment for module 2, correlating with outcome clinical parameters hospital length of stay (hosp_los) and PICU length of stay (picu_los) **(C)** Scatterplot of correlation between Gene Significance (GS) for hospital length of stay (days) *vs*. Module Membership (MM) in module 2. Pearson correlation coefficient (r) and p-values are shown. Linear regression line (purple) with a 95% confidence interval (grey) is shown. **(D)** Scatterplots of correlation between hospital length of stay (days) and normalized gene expression (CPM) of *FOXJ1, PIFO, CCDC40.* Pearson correlation coefficients (r) and p-values are shown. **(E)** Scatterplots of correlation between hospital length of stay (days) and relative cell abundance. Pearson correlation coefficients (r) and p-values are shown. P-values <0.05 are considered statistically significant and are highlighted using asterisk (*). Hosp_los = hospital length of stay (days). Picu_los, Pediatric intensive care unit (PICU) length of stay (days); HVGs, highly variable genes; WGCNA, Weighted Gene Co-Expression Network Analysis.

To investigate how the early host transcriptional response to RSV in the nasal mucosa relates to clinical characteristics, we ran weighted gene co-expression network analysis (WGCNA) on participant samples obtained within three days of hospital admission (n=28) (**Figure 3A**; **Supplemental Figures 1A, B**). We identified 13 gene modules associated with clinical parameters (**Figure 3A**). Module 2 significantly correlated with hospital and PICU length of stays (**Figure 3A**) and enriched for genes associated with ciliary processes such as *cilium movement* and *cilium organization* (**Figure 3B**). Genes identified by WGCNA with the highest correlation to hospital length of stay (*FOXJ1, PIFO, CCDC40)* are known ciliated cell genes encoding proteins required for ciliary structure and function (**Figure 3C**) (43–46). Consistent with this, expression of *FOXJ1, PIFO*, and *CCDC40* positively correlated with hospital and PICU length of stays (**Figure 3D**; **Supplemental Figure 1C**). Furthermore, in line with our previous observation that the interferon response positively correlated with viral reads (**Figure 2D**), Module 13 significantly correlated with RSV reads. This module enriched for genes associated with antiviral response processes such as *response to virus* and *type I IFN signaling* (**Figure 3A**; **Supplemental Figure 1D**). Of note, none of the modules correlated with age or sex (**Figure 3A**).

Given the association between clinical outcomes and ciliated cell genes identified by WGCNA, we performed *in silico* cell-type deconvolution on the bulk RNA-seq data to estimate the relative cellular composition in each participant sample. In line with our previous findings, there was a positive correlation between the estimated abundance of ciliated cells and hospital length of stay (**Figure 3E**). Notably, no other cell type correlated with outcomes (**Figure 3E**). Together, these data demonstrate that increased ciliated cell gene expression and estimated abundance of ciliated cells in the nasal mucosa near the time of admission are associated with worse outcomes in infants with RSV bronchiolitis.

### Epithelial gene signatures are associated with the duration of non-invasive respiratory support in critical RSV bronchiolitis

Next, we sought to investigate whether gene expression differed between infants who required NIS for a shortened (</= 3 days, n=12) or prolonged (> 3 days, n=8) length of time, despite being clinically indistinguishable at the time of sampling (**Supplemental Figure 2A**; **Supplemental Table 1**). The cut-off of 3 days was chosen empirically from the median length of NIS in our cohort (**Table 1**). Importantly, age, demographics, time from symptom onset to sample collection, level of respiratory support (flow per body weight), and supplemental oxygen were similar between the two groups (**Supplemental Table 1**). Furthermore, average IFN response gene expression, average RSV viral reads, and RSV receptor gene expression (*NCL, IGF1R*) did not differ between the shortened NIS and prolonged NIS groups (**Supplemental Figure 2B**).

**Figure 4:**
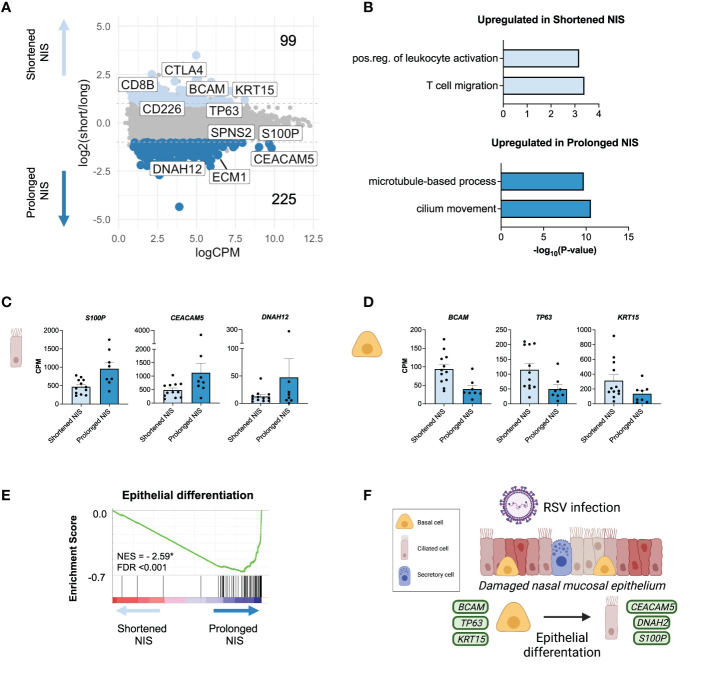
Epithelial gene signatures are associated with the duration of non-invasive respiratory support in critical RSV bronchiolitis. **(A)** MA plot of differentially expressed genes (edgeR p-value <0.05 and log2FC > |1.0| between participants requiring NIS for a shortened (</= 3 days, n = 12) or prolonged (> 3 days, n = 8) length of time. Number of differentially expressed genes upregulated (light blue) and downregulated (blue) in shortened compared to prolonged NIS are shown. **(B)** GO biological process enrichment for genes upregulated in shortened and prolonged NIS. **(C, D)** Bar graphs of gene expression (CPM) for representative up- and down-regulated genes in participants who required shortened or prolonged NIS are shown. All differentially expressed genes shown passed the cut-off as defined in MA plot in panel A: edgeR p-value<0.05 and log2FC>|1.0|, and are statistically significant. Error bars represent SEM. **(E)** Gene Set Enrichment Analysis (GSEA) enrichment plot demonstrating Epithelial Differentiation is negatively enriched in shortened compared to prolonged NIS participants. GSEA was performed on a gene list ranked by edgeR reported fold-change between shortened and prolonged NIS participant groups. Normalized enrichment score (NES) is shown. * = statistically significant FDR values <0.05. **(F)** Schematic depicting basal cell differentiation into ciliated epithelial cells to restore damaged nasal mucosal epithelium during RSV infection. Green squares represent genes expressed by the associated cell type.

A pairwise comparison between the shortened NIS and prolonged NIS groups identified 324 differentially expressed genes (**Figure 4A**). Genes upregulated in the shortened NIS group included *CD8B*, *CTLA4*, *CXCL9*, and *PRKCQ* and enriched for specific immune pathways such as *positive regulation of leukocyte activation, T cell migration*, and *TCR signaling* (**Figure 4B**; **Supplemental Figures 2C, D**). Interestingly, *CTLA4* is a negative regulator of the early innate T cell response (47, 48). *CXCL9*, also upregulated in the shortened NIS group, plays an important role in controlling viral infection (49). While the immune response to critical RSV infections is dysregulated as compared to mild or asymptomatic RSV infections, these data suggest there is a more balanced immune response in those participants who required a shortened duration of NIS compared to those who required a prolonged duration of NIS.

Genes upregulated in the prolonged NIS group enriched for ciliated cell processes, such as *microtubule-based process* and *cilium movement*, consistent with our previous observation that a ciliated cell signature correlated with worse outcomes (**Figure 4B**). The epithelium in the nose and airways contains a basal stem cell niche that can differentiate into ciliated cells upon injury to restore the epithelial barrier (50–52). *S100P, CEACAM5*, and *DNAH12* were upregulated in the prolonged NIS group (**Figure 4C**). Notably, these genes have been previously reported in a novel ciliated cell state resulting from basal cell differentiation in patients with asthma (7). In contrast, basal cell genes *BCAM, TP63*, and *KRT15* were all upregulated in the shortened NIS group (**Figure 4D**). Furthermore, genes involved in epithelial differentiation were upregulated in participants requiring prolonged NIS (**Figure 4E**). These data demonstrate that epithelial gene signatures in clinically indistinguishable infants with critical RSV bronchiolitis are associated with duration of non-invasive respiratory support (**Figure 4F**).

### Nasal transcriptome distinguishes between mild and severe critical RSV bronchiolitis

**Figure 5:**
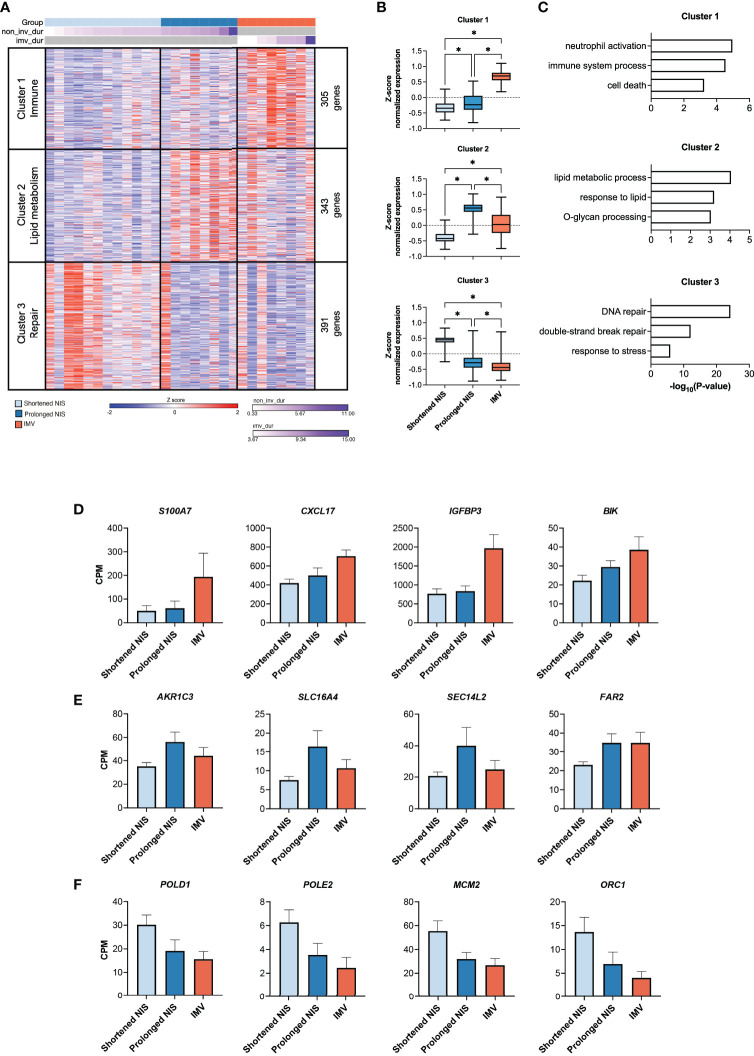
Nasal transcriptome distinguishes between mild and severe critical RSV bronchiolitis. **(A)** Heat map of 1,039 differentially expressed genes (ANOVA-like test (edgeR) p-value <0.05 between IMV (n = 8), prolonged NIS (n = 8), and shortened NIS (n = 12) participant samples. Samples are sorted by duration of respiratory support. K-means clustering identified 3 clusters characterized by Immune (Cluster 1, n = 305), Lipid metabolism (Cluster 2, n = 343), and Repair (Cluster 3, n = 391) processes. **(B)** Box plots of average z-score normalized gene expression for each group, per cluster. Error bars represent SEM. **(C)** GO biological process enrichment for Clusters 1, 2, and 3. **(D-F)** Bar graphs of normalized gene expression (CPM) for representative genes from Clusters 1, 2, and 3. Error bars represent SEM. non_inv_dur, non-invasive duration; imv_dur, invasive mechanical ventilation duration; NIS, non-invasive support; CPM, counts per million. * = p < 0.05.

To identify transcriptional differences associated with mild and severe critical RSV bronchiolitis, we compared the nasal transcriptome of participants who required IMV (n=8) to participants who required shortened (n=12) and prolonged NIS (n=8). Age, demographics, and time from symptom onset to sample collection did not differ between groups (**Supplemental Table 1**). In line with our findings in **Figure 2**, we did not observe a difference in the IFN response, viral reads, or expression of *NCL* and *IGF1R* between all three groups (**Supplemental Figure 3A**). We identified 1,039 differentially expressed genes between the IMV, prolonged NIS, and shortened NIS participant samples. Using k means clustering, we defined 3 clusters of genes characterized by Immune (Cluster 1), Lipid metabolism (Cluster 2), and Repair (Cluster 3) processes (**Figures 5A, B**). Immune cluster genes had the highest expression in participants who required IMV and enriched for immune response processes such as *neutrophil activation*, *immune system process*, and *cell death* (**Figure 5C**). Of note, there was no difference in the relative neutrophil abundance between the three groups (**Supplemental Figure 3B**). Specific genes within the Immune cluster included genes involved in neutrophil function such as *S100A7* (53, 54) and *CXCL17 (* 55), and the proapoptotic factors *IGFBP3* and *BIK* (**Figure 5D**) (56, 57). Lipid metabolism cluster genes had the highest expression in participants who required prolonged NIS (**Figures 5A, B**) and enriched for *lipid metabolic process*, *response to lipid*, and *O-glycan processing* (**Figure 5C**). Specific genes included *AKR1C3*, an aldo-keto reductase upregulated in response to oxidative stress (58), and *SLC16A4, SEC14L2*, and *FAR2*, proteins involved in lipid metabolism (**Figure 5E**) (59–61). Repair cluster genes had the highest expression in participants who required a shortened duration of NIS (**Figures 5A, B**) and enriched for *DNA repair*, *double-strand break repair*, and *response to stress* (**Figure 5C**). Specific genes included DNA polymerase subunits *POLD1* and *POLE2* (62) and components of DNA replication complexes, *MCM2* and *ORC1* (63, 64) (**Figure 5F**). Taken together, these data suggest that neutrophilic inflammation, metabolic dysregulation, and epithelial cell death are associated with disease severity in critical RSV bronchiolitis. At the same time, higher expression of genes involved in DNA repair and response to stress may promote early recovery from critical RSV infections.

## Discussion

Currently, there is no test to predict which infants admitted to the PICU with critical RSV bronchiolitis will recover quickly and which infants will require prolonged respiratory support. Using transcriptomic profiling of nasal scrape biopsies obtained within 3 days of admission to the hospital, we identified an association between nasal gene expression and clinical outcomes in infants with critical RSV bronchiolitis. Infants with a basal cell transcriptional signature required three days or less of NIS, whereas infants with a ciliated cell transcriptional signature required more than three days of NIS. Our findings suggest differences in the expression of key epithelial cell genes in the nasal mucosa reflect disease severity in children with critical RSV bronchiolitis.

The respiratory epithelium is a dynamic tissue that regenerates in response to injury (65–67). RSV primarily infects ciliated cells, resulting in the destruction of cilia and impaired mucociliary clearance (13, 68–70). Infected ciliated cells become dysmorphic, causing them to protrude and slough into the airways (11–13). This is evident early in infection, as nasal biopsies at hospital admission in children with RSV exhibit denuded epithelium with cellular projections, dysmorphic cells, and loss of integrity (68). Areas of damaged epithelium need to regenerate following injury to restore the epithelial barrier and mucociliary clearance. Regeneration includes replacing apoptotic ciliated cells through basal cell differentiation and assembly of new cilia in surviving cells (65, 66, 71). We sampled the nasal epithelium within three days of PICU admission, a point in illness when the injury is evolving (68). We observed that infants who required NIS for three days or less exhibited a basal cell transcriptional signature with increased expression of *BCAM*, *TP63*, and *KRT15*. Additionally, compared to infants who required prolonged NIS or IMV, the transcriptional profile of infants who required a shortened duration of NIS enriched for processes involved in DNA repair and response to stress. DNA repair enzymes are upregulated in response to RSV infection, and DNA repair processes promote epithelial cell survival during influenza virus infection (72–74). Altogether, this suggests that the nasal epithelium in infants who require a shortened duration of NIS is more resilient, allowing for the maintenance of the basal cell compartment and improved repair of the respiratory epithelium.

In infants who required prolonged NIS, we observed a decrease in basal cell gene expression along with an increase in ciliated cell gene expression. These data suggest that destruction of the nasal mucosa leads to differentiation of basal cells into ciliated cells in infants who required prolonged NIS. This is supported by enrichment of genes involved in epithelial cell differentiation in this group. Interestingly, we observed upregulation of *S100P, DNAH12*, and *CEACAM5* in the infants who required prolonged NIS. Recently, these genes were shown to be expressed in a unique ciliated cell state identified in the airways of individuals with asthma (7). Moreover, a recent study found that a developing ciliated cell state is associated with disease severity in COVID-19 (75). This finding is in line with our observation that a ciliated cell signature correlates with clinical outcomes in RSV. Bulk RNA-seq cannot determine whether these specific cell states are present in our cohort, thus future studies should assess their potential role in critical RSV bronchiolitis.

We observed an association between a ciliated cell transcriptional signature and hospital length of stay when analyzing the transcriptional profile of all participants sampled within three days of hospitalization. Interestingly, when we separated participants into three groups; infants who required shortened NIS, infants who required prolonged NIS, and infants who required IMV, we did not observe a ciliated cell signature in the infants requiring IMV. This would suggest the association between hospital length of stay and ciliated cell gene expression is independent of respiratory support. Indeed, some infants who receive IMV recover quickly, while some children who receive NIS have a prolonged illness. Future studies in a larger cohort of infants with critical RSV may validate a nasal transcriptional profile unique to infants who suffer a prolonged illness, irrespective of respiratory support.

Our findings suggest that upregulation of ciliary processes may reflect the degree of epithelial injury and differentiation during RSV infection. Importantly, increased epithelial injury may directly contribute to clinical severity. In pre-clinical models and autopsy specimens, RSV-induced respiratory failure is associated with obstruction of the lower airways by dysmorphic and apoptotic epithelial cells (11–13). Loss of cilia impairs mucociliary clearance, further contributing to the obstructive respiratory physiology characteristic of RSV bronchiolitis. Alternatively, the upregulation of ciliated gene expression in children who required prolonged NIS may represent differences in their baseline nasal epithelial cell populations. This interpretation would suggest that children with higher abundances of ciliated cells in the nasal mucosa prior to infection are at increased risk for prolonged illness. The samples in this study were all collected at the time of hospitalization, however future studies could investigate whether the nasal epithelial cell composition prior to infection is associated with outcomes.

There has been considerable investigation into whether viral load is associated with disease severity in RSV infection, but there has been no consensus (37–42). We did not identify an association between viral reads in the nasal mucosa and severity of illness or outcomes in our cohort. Multiple different techniques have been used to quantify viral load and multiple definitions have been used to stratify disease severity. These differences likely contribute to the variability in findings. The lack of a consistent finding across multiple studies suggests viral load in the nasal mucosa at the time of presentation does not drive disease severity or outcomes. Of note, we found that samples collected closest to symptom onset tended to have the highest viral reads as measured by RNA-seq, suggesting that viral load reflects stage of illness in infants with RSV infection.

The immune response to RSV is thought to contribute to severity of disease. Previous studies have demonstrated upregulation of the interferon response in the nasal mucosa of infants with RSV when compared to healthy or convalescent infants (17, 20). We did not observe a difference in the interferon response in the nasal mucosa between infants who required IMV and infants who required NIS. Similarly, there was no difference in the interferon response in the nasal mucosa between infants who required shortened or prolonged NIS. This is consistent with our previous observation that the nasal interferon response did not differ between children with asymptomatic to mild SARS-CoV-2 and critical RSV infections (24), suggesting the interferon response in the nasal mucosa near the time of PICU admission does not reflect disease severity. A previous study reported a difference in the magnitude of interferon signaling in the nasal mucosa in the first three days after symptom onset between infants with mild and severe disease (36). That study defined severity using a clinical score and included infants who were not hospitalized. Our study only includes infants with critical RSV bronchiolitis admitted to the PICU, which may account for the observed differences.

In the nasal transcriptional profile of the participants who required IMV, we observed enrichment for immune processes involved in neutrophil activation and degranulation. This aligns with the observation that adults with symptomatic RSV infection express genes involved in neutrophil activation (18). Further, in infants admitted to the PICU with RSV, neutrophils obtained from peripheral blood and lower airways exhibit upregulation of activation and proinflammatory genes when compared to uninfected controls (76). Neutrophils are the predominant cell type in lung tissue upon autopsy in fatal RSV infection (11, 12) and CXCL8, a primary chemokine for neutrophil recruitment, has been associated with severity of illness in RSV bronchiolitis (15, 77). While we did observe increased expression of neutrophil activation genes in our most severe group, there was no difference in estimated neutrophil abundance among our three groups. This suggests neutrophil activation near the time of admission may be a risk factor for disease severity in critical RSV bronchiolitis.

Our study had several limitations. First, the transcriptional profiles were obtained from scrape biopsies of the nasal mucosa, which contains multiple epithelial and immune cell subtypes. We applied *in silico* deconvolution to assess differences in cell abundance, however the use of complementary techniques will allow us to attribute gene expression to specific cell types, investigate rare cell types, or interrogate cell-to-cell interactions. Single-cell RNA-seq can address these areas of interest, however, certain cell types, such as neutrophils, may not tolerate sample processing and cryopreservation as well as other cell populations. Second, sample collection was performed at a single timepoint. Our goal was to define a gene signature near the time of PICU admission that was associated with outcomes, but serial sampling could provide additional insight into immune cell recruitment, epithelial cell differentiation, and the genes that drive these processes. Finally, this study focused on a small cohort of patients with critical RSV bronchiolitis requiring admission to the PICU. Validation of our findings in a larger cohort will be necessary to ascertain the predictive value of nasal gene expression in critical RSV bronchiolitis. A recent study demonstrated a correlation of nasal gene expression with a clinical severity score in non-hospitalized and hospitalized infants with RSV bronchiolitis (78). Future studies that include infants with the full spectrum of RSV disease will further inform how the host response contributes to disease severity and outcomes.

The ability to predict the duration of illness in infants with critical RSV bronchiolitis using a non-invasive nasal sample would provide invaluable prognostic information for parents and medical providers. In addition, identifying gene signatures associated with specific patient endotypes may inform the allocation of resources and development of therapeutics. To achieve this goal, it will be important to validate whether a cilia-related gene signature in the nasal mucosa during critical RSV bronchiolitis can predict the duration of respiratory support and hospitalization.

## Data availability statement

Processed, normalized counts (CPM), WGCNA modules, DEGs, and GO Processes are available in the supplementary material. Raw data has been deposited to dbGaP, accession number phs003053.v1.p1. Code is available on (https://github.com/NUPulmonary/2021_RSV_Koch).

## Ethics statement

The studies involving human participants were reviewed and approved by Institutional Review Board at Ann & Robert H. Lurie Children’s Hospital of Chicago (Chicago, IL, USA). Written informed consent to participate in this study was provided by the participants’ legal guardian/next of kin.

## Author contributions

CK, AP, and BC contributed to the conception and design of the study. CK, AP, LS, and BC contributed to acquiring, analyzing, and interpreting data and writing the manuscript. KA, HD-U, HA-V, YP, AS, JC, and GH contributed to the acquisition and analysis of data. All authors critically revised and approved the final version of the manuscript.

## Funding

This research was supported in part through the computational resources and staff contributions provided by the Genomics Compute Cluster, which is jointly supported by the Feinberg School of Medicine, the Center for Genetic Medicine, and Feinberg’s Department of Biochemistry and Molecular Genetics, the Office of the Provost, the Office for Research, and Northwestern Information Technology. The Genomics Compute Cluster is part of Quest, Northwestern University’s high-performance computing facility, to advance research in genomics. CK was supported by NIH NHLBI HL154998-01. AP was supported by the Gorter Family Foundation and NIH NHLBI 5T32HL076139-18. BC was supported by funds provided by the Manne Research Institute COVID-19 Springboard Exploratory Research Award, ATS Unrestricted Grant: Pulmonary and NIH NHLBI K08HL143127.

## Acknowledgments

The authors thank all the participants for enrolling in this study. The authors thank Rogan Grant for his help with the RNA-seq pipeline, Nikolay Markov for his help with deconvolution, and Karen Ridge for her thoughtful discussions. **Figure 4**, **Supplemental Figure 2**, and **Supplemental Figure 4** were created using BioRender.com.

## Conflict of interest

The authors declare that the research was conducted in the absence of any commercial or financial relationships that could be construed as a potential conflict of interest.

## Publisher’s note

All claims expressed in this article are solely those of the authors and do not necessarily represent those of their affiliated organizations, or those of the publisher, the editors and the reviewers. Any product that may be evaluated in this article, or claim that may be made by its manufacturer, is not guaranteed or endorsed by the publisher.

## Supplementary material

Supplementary Figure 1
**(A)** Principal component analysis (PCA) of normalized counts data (CPM) from all participant samples collected within three days of PICU admission (n=28) demonstrates a lack of structure related to clinical outcome **(B)** Heatmap of Pearson correlation of mean gene expression across all samples, hierarchical clustering performed on rows and columns. Participant samples do not cluster by respiratory support **(C)** Scatterplots of correlation between PICU length of stay (days) and normalized gene expression (CPM) of *FOXJ1, PIFO, CCDC40.* Pearson correlation coefficients (r) and p-values are shown. **(D)** GO biological process enrichment for module 13, correlating with RSV viral reads. PICU, Pediatric intensive care unit.

Supplementary Figure 2
**(A)** Nasal scrape biopsies were obtained from RSV-infected participants who were clinically indistinguishable at time of sample collection. These participants went on to require NIS for a shortened (</= 3 days, n=12) or prolonged (> 3 days, n=8) length of time. **(B)** Bar graphs of normalized gene expression (CPM) for average IFN response, average RSV viral reads, and RSV entry factor genes *NCL* and *IGF1R*. Error bars represent SEM. Log-transformed counts (log_2_CPM) are shown for viral reads for scaling purposes. **(C)** Gene Set Enrichment Analysis (GSEA) enrichment plot demonstrating T cell receptor (TCR) signaling is positively enriched in shortened compared to prolonged NIS participants. GSEA was performed on a gene list ranked by edgeR reported fold-change between shortened and prolonged NIS participant groups. Normalized enrichment score (NES) is shown. * = statistically significant FDR values <0.05. **(D)** Bar graphs of gene expression (CPM) for representative leading-edge genes, upregulated in the shortened NIS participant group. These genes were also differentially expressed genes and passed the cut-off as defined in MA plot (**Figure 4A**) and are statistically significant.

Supplementary Figure 3
**(A)** Average normalized expression of IFN response genes, RSV viral reads, and expression (CPM) of RSV entry factors *NCL* and *IGF1R* genes did not differ between participants requiring shortened NIS (n=12), prolonged NIS (n=8), or IMV (n=8). Log-transformed counts (log_2_CPM) are shown for viral reads for scaling purposes. **(B)** Relative abundance of neutrophils in participants requiring shortened NIS, prolonged NIS, or IMV. Relative abundances obtained from RNA-seq data using deconvolution (see Methods). IFN, interferon; NIS, non-invasive support; IMV, invasive mechanical ventilation.

Supplementary Figure 4 Respiratory syncytial virus is the leading cause of bronchiolitis in infants and can progress to life-threatening respiratory failure. We obtained nasal scrape biopsies within 3 days of admission from infants with critical RSV bronchiolitis. Clinical characteristics, outcome measures, and nasal mucosal gene expression were analyzed. Increased expression of ciliated cell markers and estimated ciliated cell abundance, but not immune cell abundance, was associated with prolonged need for respiratory support. In clinically indistinguishable infants, genes upregulated in those slow to recover from RSV infection enriched for epithelial differentiation. In contrast, infants who recovered more quickly exhibited upregulation of basal cell genes. Altogether, gene signatures in the nasal mucosa detected early after admission are associated with outcomes in infants infected with RSV.
