# Supplementary material for: Cilia-related gene signature in the nasal mucosa correlates with disease severity and outcomes in critical respiratory syncytial virus bronchiolitis
Source: Front Immunol. 2022 Sep 23;13:924792. doi: 10.3389/fimmu.2022.924792 (PMC9540395; doi:10.3389/fimmu.2022.924792)
Supplement: Supplementary file 1 [file Table_1.pdf]

Supplemental Table 1. Comparison of participants by respiratory support

	NIS ≤ 3 Days	NIS > 3 Days	IMV	P-Adj
**n**	12	8	8	
**Median Age (mon) [IQR]**	1.9 [1.4-2.3]	4.0 [2.0-6.0]	3.0 [1.8-5.0]	0.29
**Gest Age <35 wks at Birth (%)**	0 (0.0)	0 (0.0)	1 (12.5)	0.63
**Sex**				
Male (%)	8 (66.7)	5 (62.5)	1 (12.5)	0.18
Female (%)	4 (33.3)	3 (37.5)	7 (87.5)	
**Race**				
White (%)	5 (41.7)	6 (75.0)	4 (50.0)	0.62
African American (%)	3 (25.0)	0 (0.0)	1 (12.5)	
Asian (%)	0 (0.0)	1 (12.5)	1 (12.5)	
Other (%)	4 (33.3)	1 (12.5)	2 (25.0)	
**Ethnicity**				
Non-Hispanic (%)	8 (66.7)	8 (66.7)	6 (75.0)	0.85
Hispanic (%)	4 (33.3)	4 (33.3)	2 (25.0)	
**Sample Collection**				
Day of Illness (d) [IQR]	4.5 [3.0-6.0]	4.0 [3.0-4.0]	5.0 [3.8-5.3]	0.29
**Respiratory Support**				
Flow Rate (L/min/kg) [IQR]	1.6 [1.4-1.9]	2.1 [1.9-2.1]	-	0.29
FiO_2 (%) [IQR]	27.5 [24.0-31.3]	30.0 [28.8-36.3]	-	0.35
**Outcomes**				
Median ICU LOS (d) [IQR]	3.0 [2.7-3.5]	5.2 [4.6-5.9]	9.8 [7.8-12.0]	0.0002
Median Hospital LOS (d) [IQR]	3.8 [3.6-4.5]	6.0 [5.3-6.9]	13.2 [9.5-15.9]	0.0003
Median Respiratory Support Duration (d) [IQR]	2.67 [1.9-3.0]	4.5 [4.0-5.3]	8.5 [7.3-11.4]	0.0001

Definition of abbreviations: NIS = non-invasive support, IMV =invasive mechanical ventilation, Gest = gestational, wks = weeks, d = days, mon = months, IQR = interquartile range, L = liters, min = minute, kg = kilogram, FiO_2 = fraction of inspired oxygen ICU = intensive care unit, LOS = length of stay
